# Connections and Biases in Health Equity and Culture Research: A Semantic Network Analysis

**DOI:** 10.3389/fpubh.2022.834172

**Published:** 2022-03-29

**Authors:** Mireya Martínez-García, José Manuel Villegas Camacho, Enrique Hernández-Lemus

**Affiliations:** ^1^Department of Immunology, National Institute of Cardiology Ignacio Chávez, Mexico City, Mexico; ^2^Clinical Research Division, National Institute of Cardiology Ignacio Chávez, Mexico City, Mexico; ^3^Social Relations Department, Universidad Autónoma Metropolitana, Mexico City, Mexico; ^4^Computational Genomics Division, National Institute of Genomic Medicine, Mexico City, Mexico; ^5^Center for Complexity Sciences, Universidad Nacional Autónoma de México, Mexico City, Mexico

**Keywords:** health equity, culture, education, semantic networks, ontology

## Abstract

Health equity is a rather complex issue. Social context and economical disparities, are known to be determining factors. Cultural and educational constrains however, are also important contributors to the establishment and development of health inequities. As an important starting point for a comprehensive discussion, a detailed analysis of the literature corpus is thus desirable: we need to recognize what has been done, under what circumstances, even what possible sources of bias exist in our current discussion on this relevant issue. By finding these trends and biases we will be better equipped to modulate them and find avenues that may lead us to a more integrated view of health inequity, potentially enhancing our capabilities to intervene to ameliorate it. In this study, we characterized at a large scale, the social and cultural determinants most frequently reported in current global research of health inequity and the interrelationships among them in different populations under diverse contexts. We used a data/literature mining approach to the current literature followed by a semantic network analysis of the interrelationships discovered. The analyzed structured corpus consisted in circa 950 articles categorized by means of the *Medical Subheadings* (MeSH) content-descriptor from 2014 to 2021. Further analyses involved systematic searches in the LILACS and DOAJ databases, as additional sources. The use of data analytics techniques allowed us to find a number of non-trivial connections, pointed out to existing biases and under-represented issues and let us discuss what are the most relevant concepts that are (and are not) being discussed in the context of Health Equity and Culture.

## 1. Introduction

A first step toward an integrated understanding of social determinants and cultural issues contributing to determine the health inequity status and related issues, consists, not only in *enlisting* them, but also in sketching the interplay that these features may have among themselves to give rise to the observed impact of social constraints upon population-level health conditions (1). Social and cultural factors that are related to the inequities in health should be identified through comprehensive research and analysis (2). However, the future of health equity assessment also depends on our continued innovation in developing methods to monitor them and intervene from an integral, inclusive perspective (3–6).

Identifying social and cultural issues, aiming to determine the health inequity status across population subgroups, it has been widely discussed in recent years (7–10). Since the 1980s, there has been a growing call for nations across the globe to address health inequities (3). The past several years have been characterized by an increasing focus on solutions (11–13). Many initiatives scopes include identifying, monitoring, promoting and implementing frameworks to approach health inequities and social determinants of health (SDH) (14–19). In 2013 the WHO started a project named *Equity-oriented analysis of linkages between health and other sectors* (EQuAL) in order to identify possible approaches to the monitoring of equitable progress toward universal health coverage, centered on intersectoral barriers and identifying specific social determinants affecting health (14).

The study of health inequities itself is, however, not devoid of challenges and constraints (20). A number of factors, ranging from the social and economical conditions, to the cultural and educational background of the populations contribute to shape the panorama of health inequities, every one of these, actually a complex issue; hence there is the need for a research framework that allow to study these issues together (21). Such framework must also aim to be free from biases and allow an assessment of the matters in the most objective way possible. Alas, this is easier said than done. Along these lines, the present study intends to help us to characterize, at a large scale, the social and cultural determinants most frequently reported in current global research of health inequity and the interrelationships among them in different population and diverse contexts. To address these goals, we aim to take advantage of the vast corpus of literature already published in the PubMed/MEDLINE and other databases and investigate the research trend by applying network analysis to explore the relationships among their keywords so-called Medical Subject Headings (MeSH) remaining as unbiased as possible while doing this (22).

PubMed is the largest database of life sciences and biomedical literature in the world and is provided by the National Center for Biotechnology Information (NCBI) of the United States of America (23). PubMed's search interface implements at least five recommended search elements (reproducibility of search results, search results can be exported in full, search history, search string builder and forward citation search). PubMed/MEDLINE is indeed one of the top recommended primary sources for literature searches of peer-reviewed research in the biomedical sciences, as it possesses an extensively curated catalog (24). MEDLINE database is indexed by using MeSH terms, which are a collection of selected words or phrases that are able to represent specific concepts and form a fundamental part of the representation of knowledge (25). The MeSH dictionary is actually an *ontology*, its structure formalizes the name and definition of entities and their properties in a taxonomy-like manner able to capture conceptual interrelationships (26, 27).

Occasionally the MeSH classification includes the same term twice. One of these instances is preceded by an asterisk (^*^). Those entries allude to a MeSH term consider a major main topic of an article or a class. In the context of this study, such terms were treated as separate entities and analyzed accordingly.

The MeSH ontology has gained further relevance since recently, a number of researchers are using automated mining of scientific literature databases and network analysis as a novel methodology to know how the MeSH terms are related to each other and how their connectivity patterns helps better understand them—in terms of finding research ideas and raising or restating some hypotheses, and in summarizing a large amount of information (28). This method has also been useful for finding emergent keywords to further investigate in research areas such as immunotherapy and cancer (22, 29, 30), metabolomics (31), individual cognitive map or semantic networks (32), predictive, preventive and personalized medicine (33), biomedical sciences (34, 35), genetic (36), and other areas of health research (37–41). This will be also the approach we will follow here.

It is worth stressing that the present work is mostly of a descriptive nature. Our aim is to present the state of affairs regarding the scholarly discussion on these quite relevant subjects, to serve as a starting point for deeper analyses. In this regard, documents with the systematic searches, tables with the relationships forming the semantic networks and tables describing the topological data analytics of such networks (all of these included in the Supplementary Materials: Supplementary Tables 1–6 contain network statistics for all 6 networks discussed. Supplementary Documents 7–12 contain edge-list representations for all 6 networks discussed) will provide the readers interested in further analysis with exploration tools to navigate through the relatively extensive literature corpora on these matters.

## 2. Methods

### 2.1. Study Design

**Figure 1:**
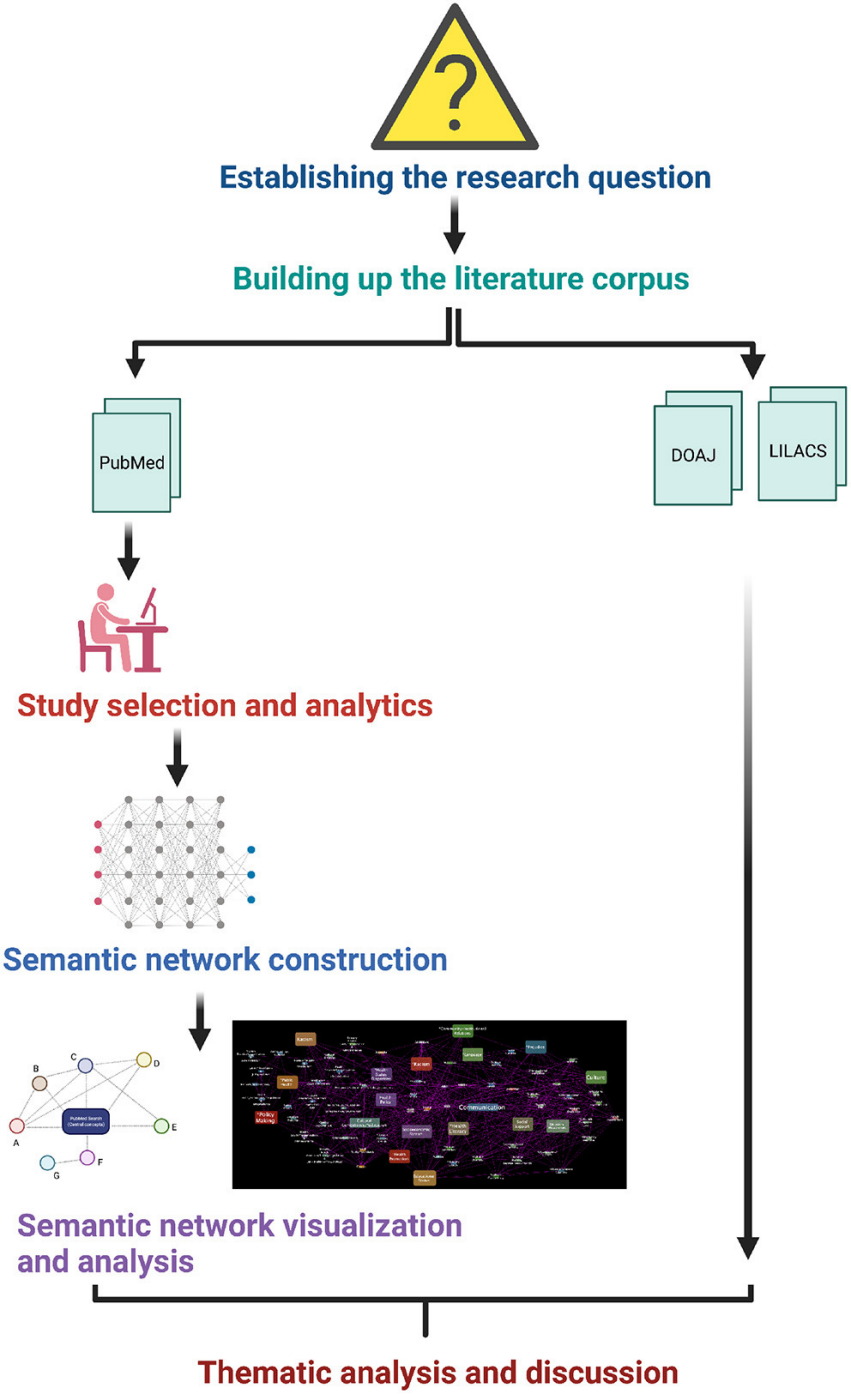
Methodological approach followed in this study. Figure created with BioRender.com.

Our methodological framework is founded on a semantic network perspective (42). Meaningful relationships among social and cultural determinants are quite difficult to unveil or highlight by resorting to traditional systematic reviews and meta-analyses that usually present the information fragmented, or at most, integrated according with the subjective appreciation of the reviewer (43, 44). Semantic network approaches to analyzing the literature have been used recently (23, 45, 46), some of them resort to computational mining of the publication databases and archives, ontology-based add context to theme-driven, systematic surveys of the literature (47–49). It is relevant to highlight that the curatorial procedures followed in this work are based on systematic and (whenever possible) objective criteria, even if it was not a completely computational curation but a hybrid approach. The methodological approach is summarized as follows (see Figure 1):

### 2.2. Stage I: Establishing the Research Questions

In recent decades there has been a growing body of evidence on equity and culture in health. Due to the complexity of each of these entities, knowledge has been accumulated in a set of seemingly disparate concepts. Under these conditions, translating knowledge into practice and improving healthcare will require a much greater effort.

In this regard, delimiting a research question in complex, multi-faceted issues such as the intricate relationships between health equity, culture and trust in the context of providing proper healthcare, that is healthcare that takes into account the situation, SDH, education and probable vulnerabilities of the populations is not an easy task. We have decided to face this issue by surveying and analyzing six different frameworks contextualized as automated literature searches used to infer semantic networks. These concepts are expected to be related and even overlapping. We believe, however, that such elections reflect somehow general aspects of this complex phenomenon in a relatively simple form.

We analyzed what are the social and cultural determinants most frequently reported in current global research of health inequity as well as the interrelationships among them. In order to delimit the scope of our work, we have chosen to focus on different aspects, aiming to present a broader (yet admittedly blurry) vision of these complex phenomena. Health equity is particularly challenged in the case of *vulnerable populations* and specially influenced by *social determinants of health*. Hence, we decided to include these two concepts within our research scope. *Culture* and *Trust* are key elements to establish human relationships that may help abridging the gap between health practitioners and healthcare users (patients, families, etc.), hence these two concepts were also considered. Since we think that a good starting point to enhance trust and establishing a proper culture is education, *health literacy* and *education* complemented the concepts we decided to analyze in this work.

In brief, in this project we have decided to investigate on **two broad research questions**:

1. What are the most frequently reported *concepts* on current global research of health equity and inequity, as represented by their associated MeSH identifiers?
2. What are the *relationships* of these concepts with issues such as the social determinants of health, vulnerable populations, culture, trust, literacy, and education in different populations and diverse contexts as captured by the published literature?

Further details on these research questions will be provided in Sections 2.6 and 3.1.

### 2.3. Stage II: Building Up a Literature Corpus

We assembled a preliminary corpus by mining the articles related to social and cultural determinants and health equity as denoted by corresponding MeSH classifiers. An automated search of the PubMed/MEDLINE database was conducted on November 16, 2021. For the purposes of this study, we used English words, Boolean AND—operator, exact phrase, and parentheses in order to group individual concepts and link them logically. Although with an individual search for a single MeSH term the results are more numerous, it is also true that a large number of documents are generated that may be related to less specific topics. After searching the databases, the documents found were imported into a data frame and duplicates were removed.

The selection criteria applied to the recovered PubMed's documents were the following:

### 2.4. Inclusion Criteria

- That each bibliographic record contain at least one MeSH term to establish the network connection between each document.
- The bibliographic record could be from any year of publication.
- The bibliographic record could be from any country of affiliation.

### 2.5. Exclusion Criteria

- The bibliographic record does not contain a title.
- The content of the bibliographic record does not coincide with the relevance of the problem under study.

We also conducted searches in the Virtual Health Library/LILACS database (https:lilacs.bvsalud.orgen) and in the Directory of Open Access Journals, DOAJ (https:doaj.org), using the same linguistic cues strategy. LILACS is a database maintained by the Latin American and Caribbean Center on Health Sciences Information (50). It includes bibliographic information from articles that have been published in a set of scientific and medical journals of the region, and that are often not covered by MEDLINE. Similar to MEDLINE, LILACS uses controlled vocabulary in indexing to ensure accurate retrieval of bibliographic references (51). DOAJ is a website that hosts a community-curated list of open access journals. DOAJ is useful as a direct search for scholarly journals across all academic disciplines. Unfortunately, there is no option to export metadata from a search (52).

### 2.6. Stage III: Study Selection and Analytics

We performed a curation of the extracted text corpus using both manual and bibliometric automated techniques (26). MEDLINE search results were saved into a plain-text Mongodb database document, then a computational literature mining procedure was performed using Python pickles to extract the information into either a corpus document or (as we will see in the upcoming Stage IV) to a network-structured file with the NetworkX Python library. The computational details of the mining strategy are sketched at the associated GitHub repository (https://github.com/CSB-IG/bibliometrics).

### 2.7. Stage IV: Data Visualization

**Figure 2:**
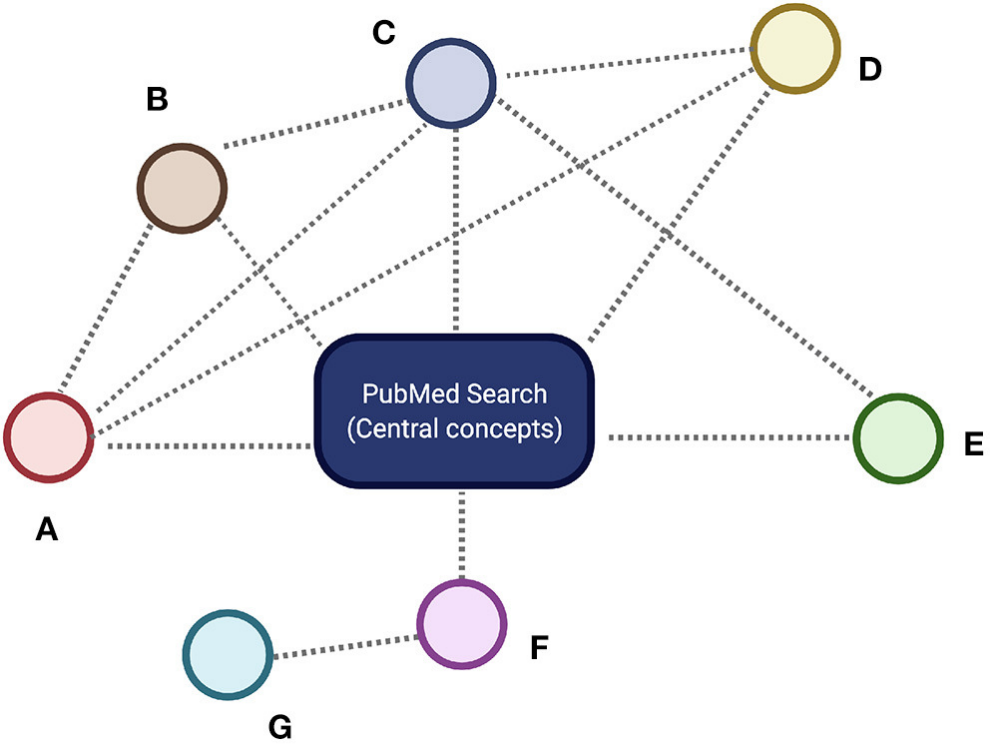
Simplified conceptual representation of a Semantic Network. The central concept or concepts (blue rectangle) are given by the main PubMed search criteria. All the articles fulfilling these search criteria are supplemented with MeSH identifiers corresponding to the different concepts. These concepts are represented by colored circles labeled **(A–G)**. Whenever two different concepts appear in the same publication in the corpus, a semantic relationship is established between them. These semantic relationships are represented by dashed lines. We can see that there are some nodes-concepts with a relatively large number of semantic connections (here nodes **A–D**) whereas others are less connected (nodes **E–G**). Well-connected nodes are deemed to be central to the concepts under discussion and are named *core* nodes in the network science terminology, whereas scarcely connected nodes are called the *periphery*. Here **(C)** is a core concept, whereas **(G)** is a peripheral concept. The hierarchy of connections of the nodes in the semantic network determines the relevance of the related concepts. Figure created with BioRender.com

Once we had a curated corpus, we built semantic networks (using co-occurrence of MeSH terms as links) and performed topological analyses of such networks to find associations between the different concepts (see Figure 2). The connectivity maps were built, so that sources and target nodes are the terms that identified the articles in corpus and a link between these nodes was drawn if two articles shared additional terms, the more terms shared, the stronger the link and hence the closer the connection of these articles were assumed. The IDs in the network construction were the PMID's of each publication. Once we have a structured corpus, -network extraction was performed with the Python code included in https://github.com/CSB-IG/bibliometrics/blob/master/mesh_network_from_medline.py, and network analysis with Python's NetworkX library(53) and Cytoscape version 2.8 (54) with the NetworkAnalyzer plugin (55). Visualization was performed using Cytoscape (56).

A first step toward the understanding of the web of interrelationships among items connected on a network is the determination of the network's local and global connectivity patterns (57). Such *topological features* as the individual and the global number of connections (called the *degree*), how are these connections assigned to the different nodes (the *degree distribution*), how important are certain nodes in the networks (called the *centrality measures*), etc. are the ones that will be used to discuss the relative importance and interplay of the different features related to our research (42). Plot visualization was also implemented to depict the main countries and dates from publications. All related source code for general text-processing may be found at https://github.com/CSB-IG/literature/tree/master/text_processing. The specific code for this work is found at https://github.com/CSB-IG/bibliometrics. Country and year mining were performed with custom-made Python scripts (articles_by_country.py and articles_by_year.py, respectively) available at https://github.com/CSB-IG/bibliometrics.

### 2.8. Stage V: Selecting, Summarizing, and Reporting the Results

As previously presented (see Section 2.2), the review questions deal with (1) **what are the** ***concepts*** **(as represented by their associated MeSH identifiers) most frequently reported in current global research of health equity and inequity** and (2) **what are their** ***interrelationships*** **with issues such as social determinants of health, vulnerable populations, culture, trust, literacy and education in different populations and diverse contexts, as presented in the published literature** as indexed in Pubmed and other health and biomedicine databases.

It is worth noticing that by building a semantic network based on a manually curated and annotated ontology (as given by the MeSH terms) on top of a comprehensive but not exhaustive database (PubMed), we are indeed introducing important assumptions. Such assumptions need to be considered as a part of our theoretical reference framework so that, all conclusions derived from this study are contingent on the validity of these assumptions (for further details on the constraints and limitations of this study see Section 4.3).

The most relevant assumptions must be summarized as follows:

1. Since only published documents indexed in the PubMed database are being retrieved for the main semantic network analysis, any contribution not indexed there (for whatever reasons) is considered **outside of our semantic universe**. This is a relevant issue since some health, biomedical or social sources, in particular in developing countries, are not indexed in PubMed. To leverage this assumption, further systematic searches were conducted in the LILACS and DOAJ databases.
2. The *concepts* here are considered based on the ontology given by MeSH classifiers. Concepts not defined as MeSH terms cannot be interpreted directly. We are aware that the MeSH ontology introduces representational biases and that other ontology used may give rise to different semantic networks.
3. All PubMed articles are manually curated and annotated with *representative* MeSH identifiers. Our computational approach relies on these annotations. Hence, if a given article was not *labeled* with a certain MeSH term, we will not count the related concept as discussed in such article, even if it is indeed touched upon.

We believe that these assumptions, still provide a general-enough framework to establish the basis for useful research, though more detailed analysis must be done to pursue a deeper understanding in more specific issues. Some of these limitations are further explored in the Section 4, in connection with other sources considered (LILACS and DOAJ).

#### 2.8.1. Thematic Analysis

We concentrated the literature corpus from the three databases reviewed. A thematic analysis of them was carried out using Atlas-ti software version 8.4.5 to identify information patterns, thus delimiting or tagging portions of a certain pre-defined category. It has been suggested that thematic analysis, is a flexible and useful research tool for identifying, analyzing and reporting patterns within data (58, 59). The theoretical approach for the analysis based on three main themes was reinforced when the literature corpus was characterized by specific codes. For the purpose of this research, the methodological strategy suggested by Terry et al. (60).

The first step, along these lines, was to organize and generate categories in the data, then analytical units were selected to assign codes and relationships to them. Subsequently, the emerging codes were contrasted with the thematic categories previously established in each of the semantic networks. Finally, three subnetworks were extracted from each of the main networks, to construct a theoretical discussion and visualize outstanding patterns of connection between various key terms. The main findings are presented in narrative form, including figures and tables in the following section.

## 3. Results

### 3.1. Stage I: Establishing the Research Question

As stated in Sections 2.2 and 2.6, the research questions guided us to investigate upon the conceptual relations between the following issues: (i) Health equity and Vulnerable Populations, (ii) Health equity and Social determinants of health, (iii) Health equity and Culture, (iv) Health equity and Trust, (v) Health equity and Health literacy, (vi) Health equity and Education.

### 3.2. Stage II: Building Up a Literature Corpus

Our automated PubMed/MEDLINE search located 950 documents. As expected, most of the articles were published in English (934/950). As previously noticed associated searches on the LILACS and DOAJ databases are also presented an used later in complementary analyses. These articles were distributed as shown in Table 1.

**Table 1:** Search results for the different databases analyzed in this work.

**Database**	**Results**	**Period**
PubMed (Medline)		
(“Health equity” [MeSH Terms]) AND (“Vulnerable Populations” [MeSH Terms])	108	2014–2021
(“Health equity” [MeSH Terms]) AND (“Social determinants of health” [MeSH Terms])	254	2015–2021
(“Health equity” [MeSH Terms]) AND (“Culture” [MeSH Terms])	127	2015–2021
(“Health equity” [MeSH Terms]) AND (“Trust” [MeSH Terms])	14	2015–2021
(“Health equity” [MeSH Terms]) AND (“Health literacy” [MeSH Terms])	27	2015–2021
(“Health equity” [MeSH Terms]) AND (“Education” [MeSH Terms])	420	2014–2021
Virtual Health Library (LILACS)		
“Health equity” AND “Vulnerable Populations”	19	2008–2021
“Health equity” AND “Social determinants of health”	57	2005–2021
“Health equity” AND “Culture”	27	2003–2021
“Health equity” AND “Trust”	4	2008–2019
“Health equity” AND “Health literacy”	2	2011–2021
“Health equity” AND “Education”	135	1992–2021
DOAJ		
“Health equity” AND “Vulnerable Populations”	93	2004–2021
“Health equity” AND “Social determinants of health”	304	2007–2021
“Health equity” AND “Culture”	249	2000–2021
“Health equity” AND “Trust”	289	2006–2021
“Health equity” AND “Health literacy”	196	2004–2021
“Health equity” AND “Education”	1,817	1995–2021

Interestingly for such related frameworks only 235 out of 950 documents (24.7 %) were overlapped between the different searches.

### 3.3. Stage III: Study Selection and Analytics

Semantic networks were constructed from the data mining of the different sub-corpora (corresponding to the different literature surveys). Some descriptive results for each of these surveys will be presented next.

We used Atlas-ti to perform thematic analysis to identify patterns of meaning across searches. Three themes emerged from the analysis: (1) identify social and cultural determinants of health inequity, (2) targeted populations and (3) Modalities of social and cultural response in various contexts (scientific, academic, political, governmental, among others).

Additional keywords to further investigate emerged from the analysis: SARS-CoV-2, Professional-patient relations, Patient acceptance of Health Care, Health Promotion, Cultural competency, Social support, Health knowledge, Attitudes and practice, Health communication and Communication barriers. These issues are considered in detail in the Section 4 (see Section 4.2).

#### 3.3.1. Health Equity and Vulnerable Populations Network

**Figure 3:**
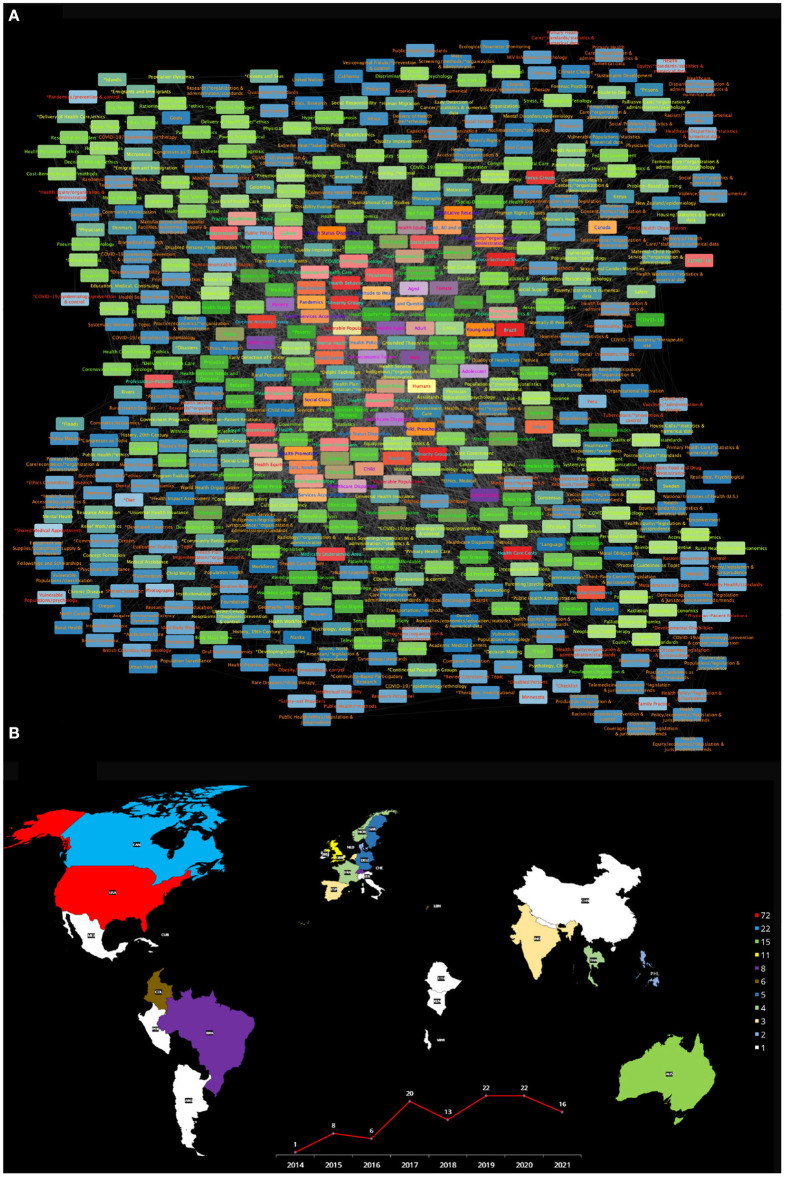
**(A)** Semantic network built from the search Health equity [MeSH Terms] AND Vulnerable Populations [MeSH Terms]: from 2014 to 2021. **(B)** The publishing countries and number of entries. USA, United States of America; AN, Canada; AUS, Australia; GBR, United Kingdom; BRA, Brazil; CHE, Switzerland; COL, Colombia; LBN, Lebanon; DEU, Germany; SWE, Sweden; FRA, France; NOR, Norway; THA, Thailand; IND, India; NLD, Netherlands; ESP, Spain; DNK, Denmark; PHL, Philippines; ARG, Argentina; CHI, Chile; CHN, China; CUB, Cuba; ETH, Ethiopia; IRL, Ireland; ITA, Italy; JAM, Jamaica; KEN, Kenya; MWI, Malawi; MEX, Mexico; NPL, Nepal; PER, Peru.

This network (see Figure 3) included 551 nodes (MeSH terms or *concepts*, the basic semantic units) and 6,010 edges, corresponding to the semantic relationships (*k*) between the nodes-concepts (see Methods). The more connected term, as expected, was *Human* with 550 semantic relationships in this network. This will be the case for all of the studies considered since all human health research in PubMed is labeled with this term. *Vulnerable populations* and *Health equity* (on their two forms), being the basis of our search were also among the most central concepts with 365 (*Vulnerable Populations*), 294 (*Health Equity), 180 (*Vulnerable Populations) and 168 (*Health Equity*) semantic relationships, respectively. Aside from demographic classifiers –*Female* (*k* = 163), *Male* (*k* = 133), *United States* (*k* = 123) and others that, as in the case of *Human* are standard or de facto MeSH classifiers in human health research– *SARS-CoV-2* emerged as an important concept in the discussion with 120 semantic relationships in the network. This is, of course, consistent with the ongoing pandemic, but also reflects the fact that this pandemic has evidenced a number of health disparity issues in vulnerable populations (61, 62).

The following relevant concepts in the scholarly literature discussion on Health Equity and Vulnerable populations point out to known issues. Concepts such as *Middle Aged* (*k* = 118), *Poverty* (*k* = 117), *Healthcare disparities* (*k* = 104), *Socioeconomic factors* (*k* = 96), *Adolescent* (*k* = 94), *Aged* (*k* = 91), *Healthcare disparities (*k* = 89), *Adult* (*k* = 82), *Child* (*k* = 75), *Health Services Accesibility* (*k* = 72) and Health Equity/*organization & administration (*k* = 69). We have presented here the Top20 more connected concepts in this semantic network. For the full list, please refer to Supplementary Document 7, network topology statistics for this network can be found in Supplementary Table 1.

Close examination of concepts such as *Poverty* within this network reveals important relationships with issues such as *Attitude to health, Health behavior, Professional-patient relations* and *Patient acceptance of Health Care*. For *Healthcare disparities*, in turn, related topics associated with the role of culture and education included *Attitude to health* and *Health behavior* but in this case there was no published literature linking *Professional-patient relations*, nor *Patient acceptance of Health Care*.

Furthermore, in the discussion of health equity and vulnerable populations in the health professional literature (as captured by this network), known vulnerable populations are not actually *central* to the discussion. Semantic relevance is often captured, not only by the degree centrality, but also by the importance *rank* (R) in the degree distribution. The most connected concept of a given network has rank 1, the second most connected concept has rank 2, and so on. Henceforth, we will often refer to the relevance of a given concept by stating its connectivity degree and rank (*k*,*R*). For instance, in this network, we can find less central concepts such as *Social Justice* (*k* = 47, *R* = 37) and *Racism* (*k* = 47, *R* = 38). Followed by *Minority groups* (*k* = 42, *R* = 42), *Hispanic Americans* (*k* = 34, *R* = 59), *Homeless persons* (*k* = 25, *R* = 88), *Disabled persons* (*k* = 24, *R* = 104), and *Refugees* (*k* = 24, *R* = 106). *Sexual and Gender Minorities* (*k* = 13, *R* = 313), *Transients and Migrants* (*k* = 12, *R* = 337) and *Prisoners* (*k* = 8, *R* = 474) are indeed significantly relegated in this discussion. Other vulnerable populations such as *African Americans, American Indians or Alaska Natives, Persons with Mental Disabilities* are not even represented in this *comprehensive* survey of the literature.

Let us now take a look at the role that *Culture, Education* and related concepts are playing in this discussion (as represented by the semantic network). The first (somewhat) related concepts that appear are *Health Promotion* (*k* = 57, *R* = 26) and *Attitude to health* (*k* = 52, *R* = 29), followed by *Patient acceptance of healthcare* (*k* = 33, *R* = 62), *Culture* (*k* = 33, *R* = 63), *Health education, dental* (*k* = 21, *R* = 144), *Cultural diversity* (*k* = 21, *R* = 151), and *Health literacy* (*k* = 21, *R* = 165). Also concepts such as *Cultural competency* (*k* = 18, *R* = 215) and even *Language* (*k* = 11, *R* = 356) that may potentially contribute to both health inequities and population vulnerabilities are notably misrepresented.

In Figure 3B, further details about the countries of origin of the publications that formed the corpus for this semantic network (for *Health Equity* and *Vulnerable populations*) (color-coded according with the number of publications generated by each *country*), as well plot presenting the number of articles produced each year from 2014 to 2021 are presented. It can be noticed that a relatively small number of countries contribute to the discussion on these matters, and that many of them are either developed countries or emerging economies. We can also highlight the fact that there is a relatively low number of works discussing *Health Equity* and *Vulnerable populations*, with no more than 22 articles published within a given year.

In brief, the **Health equity and Vulnerable populations** semantic network, as comprehensively curated from the PubMed database, presents some important general tendencies, but also evidences some remarkable biases and misrepresentations (particularly, underrepresenting a number of relevant concepts). On the one hand, we have seen that the network reflects the importance of sociodemographics for the healthcare of vulnerable populations and highlights some health disparities that have become evident with the onset of the COVID-19 pandemic. It also shows that organizational and administrative issues have been at the core of the scholarly discussion on these matters. On the other hand, however, there is a noteworthy underrepresentation of concepts that can be considered relevant for the discussion on health equity and vulnerable populations, such as *Social justice, Racism, Minorities, Migrants, Homeless persons, Sex and gender minorities, Cultural diversity* and *Language* to name but a handful. Other important issues are not only relegated but absolutely absent from the discourse (as captured by this network), among these we can mention *African Americans, American Indians or Alaska Natives, Persons with Mental Disabilities*. By recognizing the worth of the discussed concepts, as well as the shortcomings and biases in other relevant issues, it will be possible to work toward a more equitable scholarly dialogue on the many dimensions of the health equity and vulnerable populations problem.

It is worth noticing that further exploration of the intricate web of relationships, perhaps with particular questions in mind, may be performed by *navigating* the interactive networks. The use of visual tools such as Cytoscape or iGraph is recommended, but the Supplementary Network Documents are also stand-alone searchable.

#### 3.3.2. Health Equity and Social Determinants of Health Network

**Figure 4:**
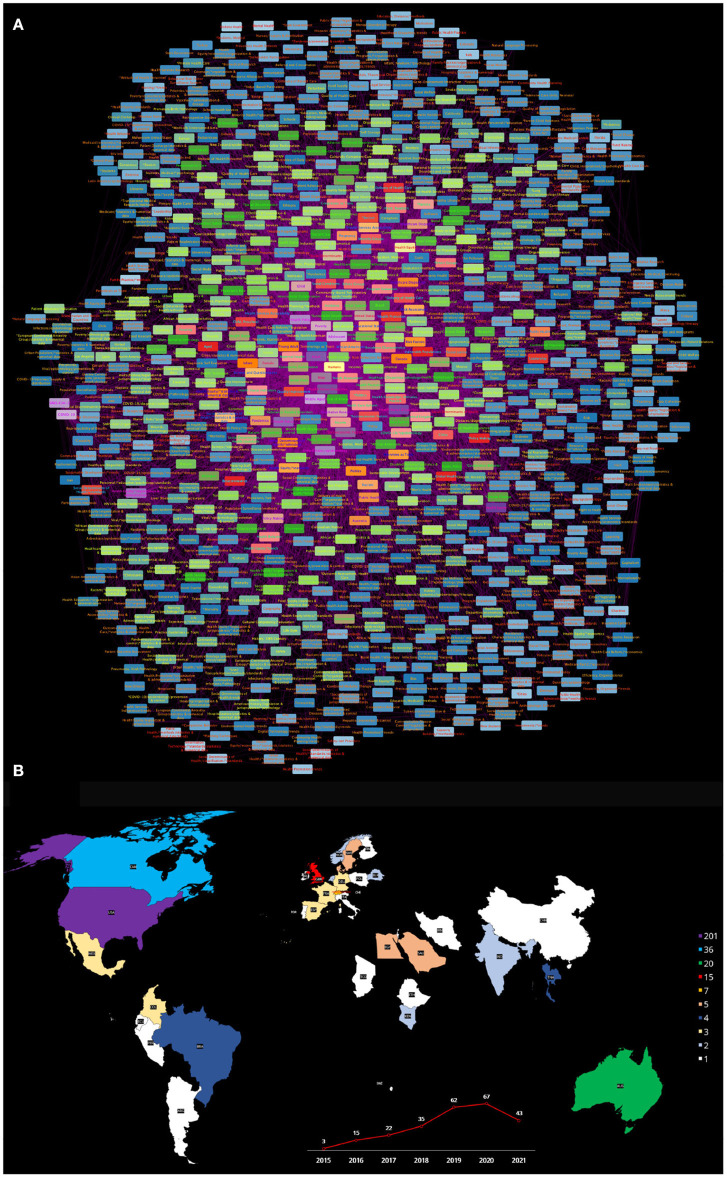
**(A)** Semantic network built from the search Health equity [MeSH Terms] AND Social determinants of health [MeSH Terms]: from 2015 to 2021. **(B)** The publishing countries and number of entries. USA, United States of America; CAN, Canada; AUS, Australia; GBR, United Kingdom; CHE, Switzerland; DNK, Denmark; EGY, Egypt; SAU, Saudi Arabia; SWE, Sweden; BRA, Brazil; THA, Thailand; CHI, Chile; COL, Colombia; FRA, France; DEU, Germany; MEX, Mexico; ESP, Spain; BLR, Belgium; IND, India; KEN, Kenya; NLD, Netherlands; NOR, Norway; ARG, Argentina; TCD, Chad; CHN, China; ECU, Ecuador; ETH, Ethiopia; FIN, Finland; IRN, Iran; IRL, Ireland; ISR, Israel; ITA, Italy; MLT, Malta; PER, Peru; POL, Poland; PRT, Portugal; SWZ, Swaziland.

This network (see Figure 4) is composed of 921 nodes and 10,156 semantic relationships. As in the case of the previous network, the higher ranked concepts in this semantic network referred to *Humans* (*k* = 912), followed by *Health Equity* (*k* = 519), *Social Determinants of Health* (*k* = 459), *Social Determinants of Health (*k* = 378) as well as demographic items: *United States* (*k* = 294), *Female* (*k* = 280) and *Male* (*k* = 244). Subsequent relevant concepts in the semantic network connectivity structure are *Socioeconomic factors* (*k* = 241, *R* = 8), followed by *Health Status Disparities (*k* = 230, *R* = 9), *Health equity* (*k* = 206, *R* = 10), *Health Status Disparities* (*k* = 177, *R* = 11), *Adult* (*k* = 171, *R* = 12), *Health policy* (*k* = 154, *R* = 13), Health Equity/*organization & administration (*k* = 137, *R* = 14), *United States/epidemiology* (*k* = 137, *R* = 15), *Health Policy (*k* = 133, *R* = 16), *SARS-CoV-2* (*k* = 128, *R* = 17), *Middle Aged* (*k* = 118, *R* = 18); as well as *Adolescent* (*k* = 110, *R* = 19) and *COVID-19* (*k* = 110, *R* = 20) to complete the Top20 concepts of this semantic network. For the full list, please refer to Supplementary Document 8, network topology statistics for this network can be found in Supplementary Table 2.

Among well-known SDH, *Poverty* (*k* = 103, *R* = 21), later on come *Educational status* (*k* = 65, *R* = 40), *Social conditions* (*k* = 45, *R* = 63), *Health knowledge, attitudes and Practice* (*k* = 42, *R* = 70), *Residence characteristics* (*k* = 41, *R* = 71), and *Housing* (*k* = 40, *R* = 72), then *Income* with (*k* = 38, *R* = 86) and further down the list come *Social class* (*k* = 35, *R* = 91) and *Social support* (*k* = 34, *R* = 98), that are relatively low ranked (91 and 98 out of 921) in spite of being considered among the more relevant SDH.

In relation to concepts related to *Culture* and *Education*, aside from *Educational status*, again we see that these topics are not central to the current discussion (as proxied by this semantic network). *Culture* (*k* = 24, *R* = 157) for instance is somehow relegated in this the network. With related topics such as *Culturally competent care (*k* = 19, *R* = 247), *Organizational culture* (*k* = 19, *R* = 248), *Cultural Diversity* (*k* = 18, *R* = 266) and Cultural competency/*education (*k* = 17, *R* = 282) even less central to the discussion. Regarding education, the network includes concepts such as *Early intervention, educational* (*k* = 31, *R* = 113), Patient advocacy/*education (*k* = 19, *R* = 239), *Public Health/education* (*k* = 13, *R* = 453), Minority Health/*education (*k* = 12, *R* = 507). These low relevance ranks confirm the fact that the discussion about these matters is lagging with respect to other issues, more central in the current literature on Health Equity and the SDH.

This network reveals that known SDH are being discussed in relation to *Culture* and *Education* issues. We already commented on the published literature discussion in connection to *Poverty*. Other social determinants observed in this semantic network such as *Health knowledge, attitudes and practice* have been discussed in relation to Social determinants of health/*ethnology and *Educational status* however *Culture* as such is somehow absent in the scholarly discussion, that apparently is centered in ethnic features of the populations rather than on cultural issues. A more detailed and careful examination of the literature is however needed before making any conclusion in this regard.

As in the case of the health equity and vulnerable populations, the semantic network representing the interrelationships of **Health equity and Social determinants of health** presents a picture of the scholarly discourse with some nuances. It is a larger network with more than 900 concepts and 10,000 semantic relationships. Demographics and search terms are again dominant in the discourse, as expected. Concepts related to administrative and organizative aspects (*Health policy, Health equity/organization and administration, United States Epidemiology*) are, one more time, central to the discourse. A bit downgraded are concepts such as *Poverty, Educational status, Housing*, and *Residence characteristics*, in spite of being relevant components of the SDH problem. However, flagrant underrepresentation can be noticed in the case of item related to culture, being ranked between the 247th and the 507th concepts out of 921, with *Culture* not even incorporated into the discourse on Health equity and Social determinants of health.

As in the case of the previous network, Figure 4B presents a map with the different countries contributing to the published literature on *Health equity* and *Social determinants of health*, as well as a plot of the number of works published every year since 2015 on these issues. The main countries contributing to the scholarly discussion on these issues are quite similar to the one in the previous network. This is relevant for a global view of these matters, since it is likely that the academic view on the matters may be biased due to peculiarities of the health systems of these countries.

#### 3.3.3. Health Equity and Culture Network

This network consists of 691 nodes-concepts and 7,836 edges-relationships. Relevant concepts include *Humans* (*k* = 684), *Health equity (*k* = 340), *Female* (*k* = 310), *Health Equity* (*k* = 253), *Male* (*k* = 246) ranked 1st to 5th. *Cultural diversity* comes 6th with (*k* = 192), followed by *United States* (*k* = 191), *Cultural diversity (*k* = 169), *Cultural competency* (*k* = 163) and *Culture* ranked in 10th (*k* = 161). The rest of the Top20 concepts are *Adult* (*k* = 138), *Socioeconomic factors* (*k* = 130), *Middle aged* (*k* = 122), *Qualitative research* (*k* = 106), *Cultural competency (*k* = 97), *Young adult* (*k* = 94), *Surveys and questionnaires* (*k* = 93), *Healthcare disparities (*k* = 90), *SARS-CoV-2* (*k* = 80), and Health equity/*standards (*k* = 76), respectively.

Other concepts related to culture that appear in this network are comparatively lagged behind, such is the case of *Cultural characteristics* (*k* = 53, *R* = 41), *Organizational culture* (*k* = 53, *R* = 42), Cultural competency/*education (*k* = 49, *R* = 47), *Paternalism* (*k* = 39, *R* = 63), *Culture (*k* = 32, *R* = 88), *Culturally competent care (*k* = 31, *R* = 93). Furthermore, *Language* (*k* = 29, *R* = 103) a central aspect of culture and *Organizational culture (*k* = 25, *R* = 121) which are relevant for healthcare policy, design and practice are also less central to the discourse than expected. We can also mention –in connection to culture– *Religion* (*k* = 24, *R* = 131), Culturally competent care/*organization & administration (*k* = 20, *R* = 170) and *Cross-cultural comparison (*k* = 19, *R* = 214). The rest of the concepts are presented in Supplementary Document 9, network topology statistics for this network can be found in Supplementary Table 3.

Although this network seems to be less biased that other semantic graphs analyzed, it remains worrying that concepts such as *Empathy* (*k* = 13, *R* = 443), *Self-concept* (*k* = 11, *R* = 530) and *Attitude to health/ethnology* (*k* = 10, *R* = 564), that we consider to be central to understand how culture contributes to shaping health equity (or inequities), remain somehow low ranked in the literature's discourse on the matters.

#### 3.3.4. Health Equity and Trust Network

This is a relatively smaller semantic network consisting in 140 nodes-concepts and 1433 edges. Main concepts regarding network centrality degree are as follows (Top20 ordered according to their ranking) *Humans* (*k* = 139), *Trust* (*k* = 93), *Male* (*k* = 63), *Health equity* (*k* = 63), *Aged* (*k* = 57), *Middle Aged* (*k* = 57), *Health equity (*k* = 54), *Communication* (*k* = 51), *Qualitative research* (*k* = 46), *Trust (*k* = 46), *Female* (*k* = 40), *Adult* (*k* = 40), *Social support* (*k* = 28), Pulmonary disease, Chronic obstructive/*rehabilitation (*k* = 28), *Saskatchewan* (*k* = 28), *Patient Acceptance of health care (*k* = 28), *Shame* (*k* = 28), *Self-Management* (*k* = 28), *Case management* (*k* = 28) and *Disease management* (*k* = 28).

Other related items are *Attitude to health (*k* = 28), *Focus groups* (*k* = 28) and *Patient participation* (*k* = 28). Followed further down in relevance by concepts like *Stakeholder participation* (*k* = 22, *R* = 37), *Trust/psychology* (*k* = 18, *R* = 52) and *Culture* (*k* = 14, *R* = 83). Issues such as *Health communication (*k* = 9, *R* = 125) and *Communication barriers (*k* = 7, *R* = 134) that are instrumental for a balanced discussion of trust are also somehow disregarded. It calls into attention that a number of terms related to organizational and administrative aspects are included in (and central to) the discussion (as proxied by the semantic network), but fewer aspects of a personal or emotional side of the issue are included.

For the full list, please refer to Supplementary Document 10, network topology statistics for this network can be found in Supplementary Table 4.

#### 3.3.5. Health Equity and Health Literacy Network

The Health equity and Health literacy network is conformed by 166 concepts (nodes) and 1,670 semantic relationships or edges. The more connected components are *Humans* (*k* = 165), *Health equity (*k* = 110), *Health literacy (*k* = 97), *Health literacy* (*k* = 86), *Health equity* (*k* = 78), *Adult* (*k* = 69), *Male* (*k* = 66), *Female* (*k* = 58), *United States* (*k* = 47), and *Middle aged* (*k* = 46) that appears in the first 10 positions respectively. Concepts ranked from the 10th to the 20th are as follows: *Qualitative research* (*k* = 39), *Aged* (*k* = 37), *Social support* (*k* = 37), *Health services accessibility (*k* = 36), *Social determinants of health* (*k* = 36), *Australia* (*k* = 30), *Adolescent* (*k* = 30), *Public health* (*k* = 30), Pulmonary disease, Chronic obstructive/*rehabilitation (*k* = 28) and *Saskatchewan* (*k* = 28).

Other relevant concepts that appear on this network are *Attitude to health (*k* = 28), *Patient participation* (*k* = 28), *Patient education as topic* (*k* = 28), *Culture* (*k* = 24) and *Health promotion* (*k* = 22). Interestingly *Literacy* (*k* = 16, *R* = 75) and *Reading* (*k* = 16, *R* = 80) appear in unexpectedly low key positions. *Communication barriers* (*k* = 14, *R* = 93) comes still later on, aside with *Educational status* (*k* = 14, *R* = 94) and *Culture* (*k* = 14, *R* = 95) and *Health knowledge, attitudes, practice* (*k* = 12, *R* = 112). It seems that the emphasis on health literacy in the current discourse is in relation to literacy in the healthcare professionals and not so much about health literacy in the general population. The recent *infodemic* around COVID-19 has further highlighted the strong urgency for the general population to be more literate on healthcare and public health issues.

For the full list, please refer to Supplementary Document 11, network topology statistics for this network can be found in Supplementary Table 5.

#### 3.3.6. Health Equity and Education Network

**Figure 5:**
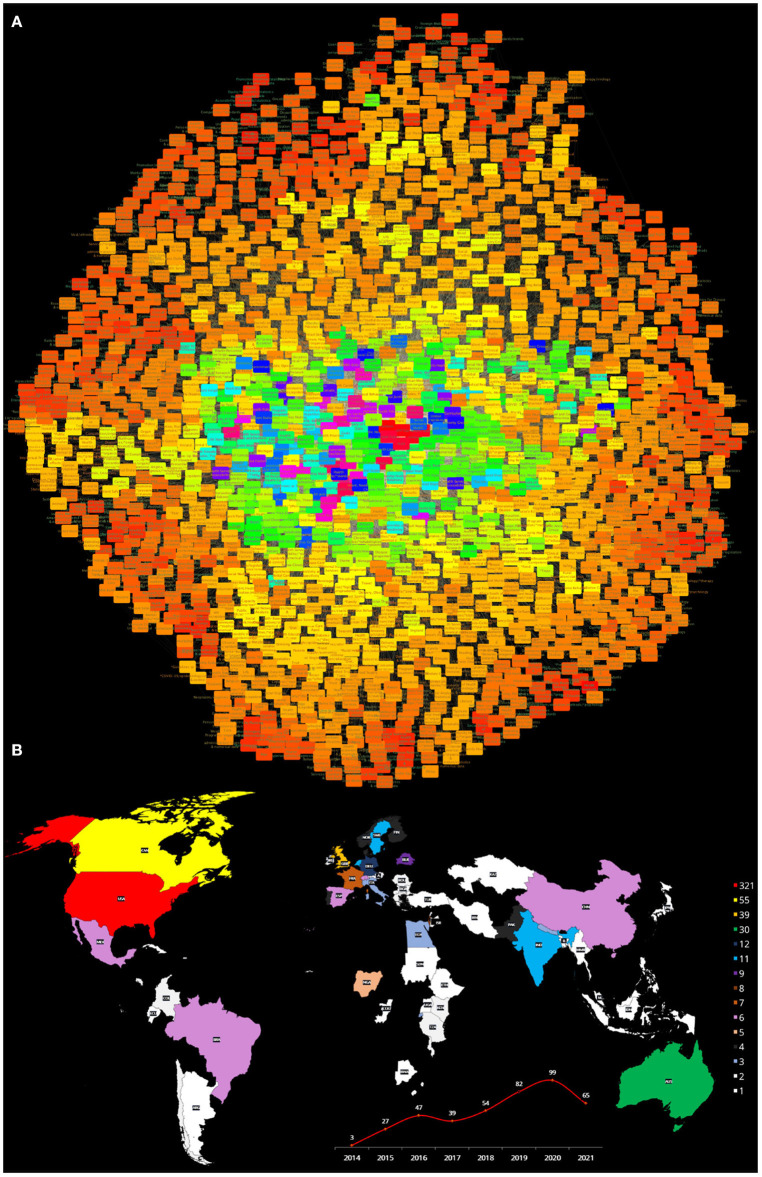
**(A)** Semantic network built from the search Health equity [MeSH Terms] AND Education [MeSH Terms]: from 2014 to 2021. **(B)** The publishing countries and number of entries. USA, United States of America; CAN, Canada; GBR, United Kingdom; AUS, Australia; DEU, Germany; IND, India; NLD, Netherlands; SWE, Sweden; BLR, Belgium; ISR, Israel; FRA, France; BRA, Brazil; CHN, China; MEX, Mexico; ESP, Spain; CHE, Switzerland; NGA, Nigeria; DNK, Denmark; FIN, Finland; NOR, Norway; PAK, Pakistan; PRT, Portugal; EGY, Egypt; ITA, Italy; NPL, Nepal; RWA, Rwanda; AUT, Austria; CHL, Chile; COL, Colombia; CRI, Costa Rica; GRC, Greece; IRL, Ireland; JOR, Jordan; KEN, Kenya; LBN, Lebanon; MWI, Malawi; ROU, Romania; TZA, Tanzania; ARG, Argentina; BGD, Bangladesh; BWA, Botswana; BGR, Bulgaria; COG, Congo; CUB, Cuba; ECU, Ecuador; ETH, Ethiopia; GMB, Gambia; IDN, Indonesia; IRN, Iran; JPN, Japan; KAZ, Kazakhstan; LSO, Lesotho; MKD, Macedonia; MYS, Malaysia; MLT, Malta; MMR, Myanmar; PHL, Philippines; PRI, Puerto Rico; SGP, Singapore; SDN, Sudan; SWZ, Swaziland; TUR, Turkey; UGA, Uganda.

This semantic graph (see Figure 5) consists of 1,673 nodes (MeSH terms) and 21,952 edges-relationships among them. Most connected concepts were: *Humans* (*k* = 1659), *Health equity (*k* = 853), *Female* (*k* = 737), *Health equity* (*k* = 602), *Male* (*k* = 570), *United States* (*k* = 505), *Adult* (*k* = 475), *Middel aged* (*k* = 321), *Socioeconomic factors* (*k* = 475) and *Health promotion* (*k* = 271), ranked in the first 10 places, respectively. The next 10 more connected concepts were: *Surveys and questionnaires* (*k* = 2641), *Aged* (*k* = 254), *Needs assessment* (*k* = 241), Health equity/*organization & administration (*k* = 235), *Health promotion (*k* = 231), *Child* (*k* = 227), *Adolescent* (*k* = 218), *Health status disparities* (*k* = 210), *Qualitative research* (*k* = 208) and *Young adult* (*k* = 206).

Other MeSH terms related to education within this semantic network, aside from *Educational status* (*k* = 200, *R* = 21), calls to attention that other concepts, essential to understand the role of education in health equity are placed in less relevant positions in the semantics of the scholarly discussion, such is the case of *Health literacy (*k* = 97, *R* = 53), *Health literacy* (*k* = 86, *R* = 63), *Cultural competency* (*k* = 79, *R* = 70), *Universities* (*k* = 73, *R* = 78), as well as *Health knowledge, attitudes, practice* (*k* = 66, *R* = 91), *Patient education as topic* (*k* = 65, *R* = 94), *Education, medical (*k* = 62, *R* = 101), as well as its counterpart *Education, medical* (*k* = 57, *R* = 112). Additional concepts have even lower connectivity degrees, such as *Healt education (*k* = 41, *R* = 169), *Culture (*k* = 32, *R* = 258), *Learning* (*k* = 32, *R* = 259), *Knowledge* (*k* = 29, *R* = 293), as well as Health education/*ethics (*k* = 24, *R* = 382). Further down the list come *Access to information (*k* = 19, *R* = 573) and *Mothers/education (*k* = 19, *R* = 594). These latter concepts are strikingly underrepresented: *how can one envision health equity through education when access to information is ranked 573 in the list of relevant concepts and mothers' education comes in place 594?*

The fact that this semantic network is relatively large and somehow rich in terms, may be connected with a nascent interest in the role of education in the context of health equity, which is, in itself, remarkable. As in other networks discussed, however, it seems that technical and administrative issues are dominant, whereas issues more closely related to the individuals and populations are somehow relegated or even absent. Again, as it can be seen in Figure 5B, there may be a representation bias in the discussion toward the situation in the countries that are contributing to this discourse.

For the full list, please refer to Supplementary Document 12, network topology statistics for this network can be found in Supplementary Table 6.

### 3.4. Stage IV: Data Visualization

Data visualization is indeed a relevant component of network analytics. Visual display provides a helpful overview of the structure of complex networks. Since network depictions are indeed aimed at being *representational*, looking at their full structure allow us to generate conceptual maps. This is all the more relevant when the network themselves encode conceptual information such as the case of semantic networks (23, 45, 46).

In this section, we will provide a general schematic view of the semantic networks analyzed in this work as well as three representative examples. As previously mentioned the information to build all of the networks (not just the three shown here) is given in the Supplementary Materials.

#### 3.4.1. Feature Specific Subnetworks

**Figure 6:**
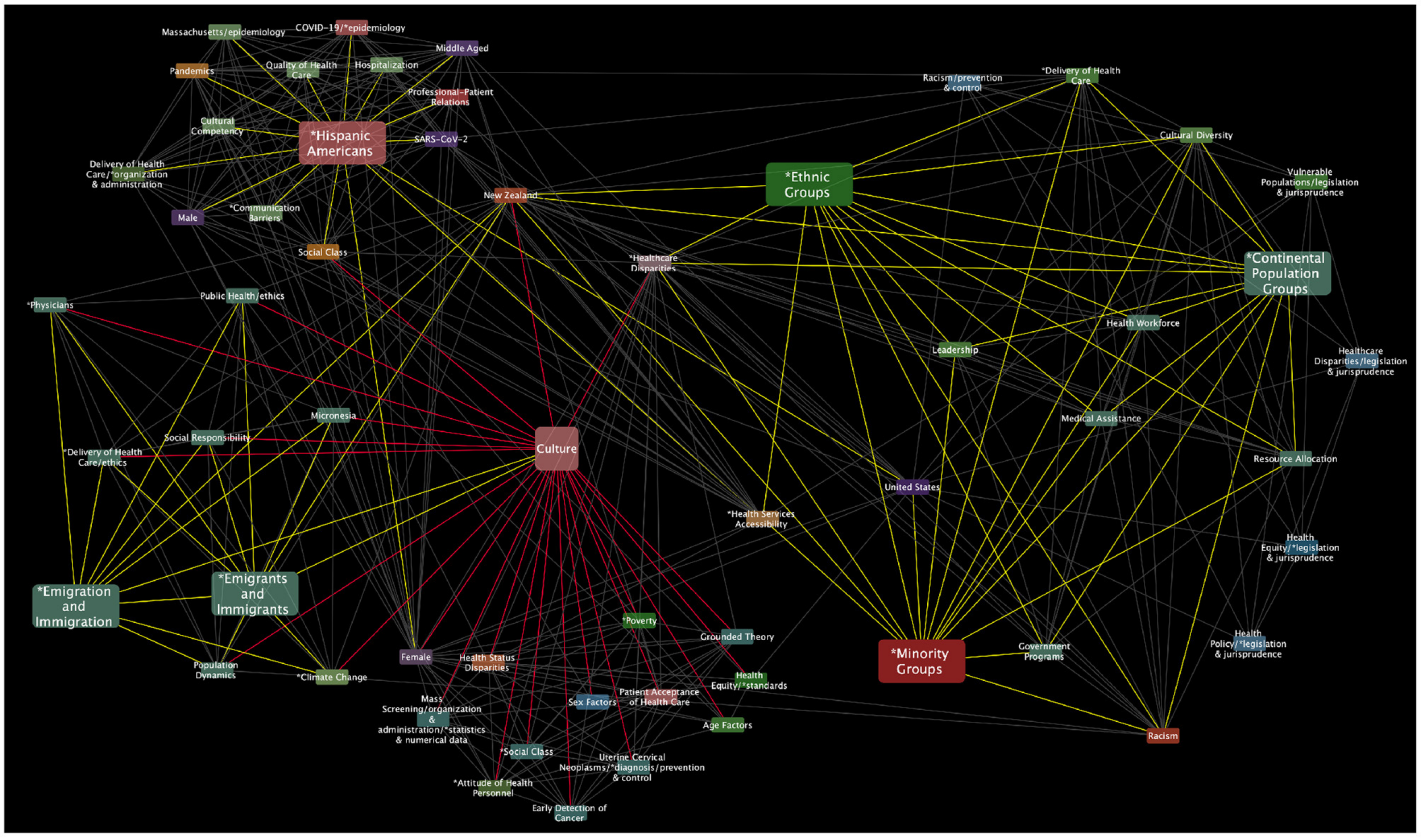
Health equity, Vulnerable Populations, and Culture MeSH terms subnetwork. This *HVC subnetwork* has 57 nodes and 381 edges.

**Figure 7:**
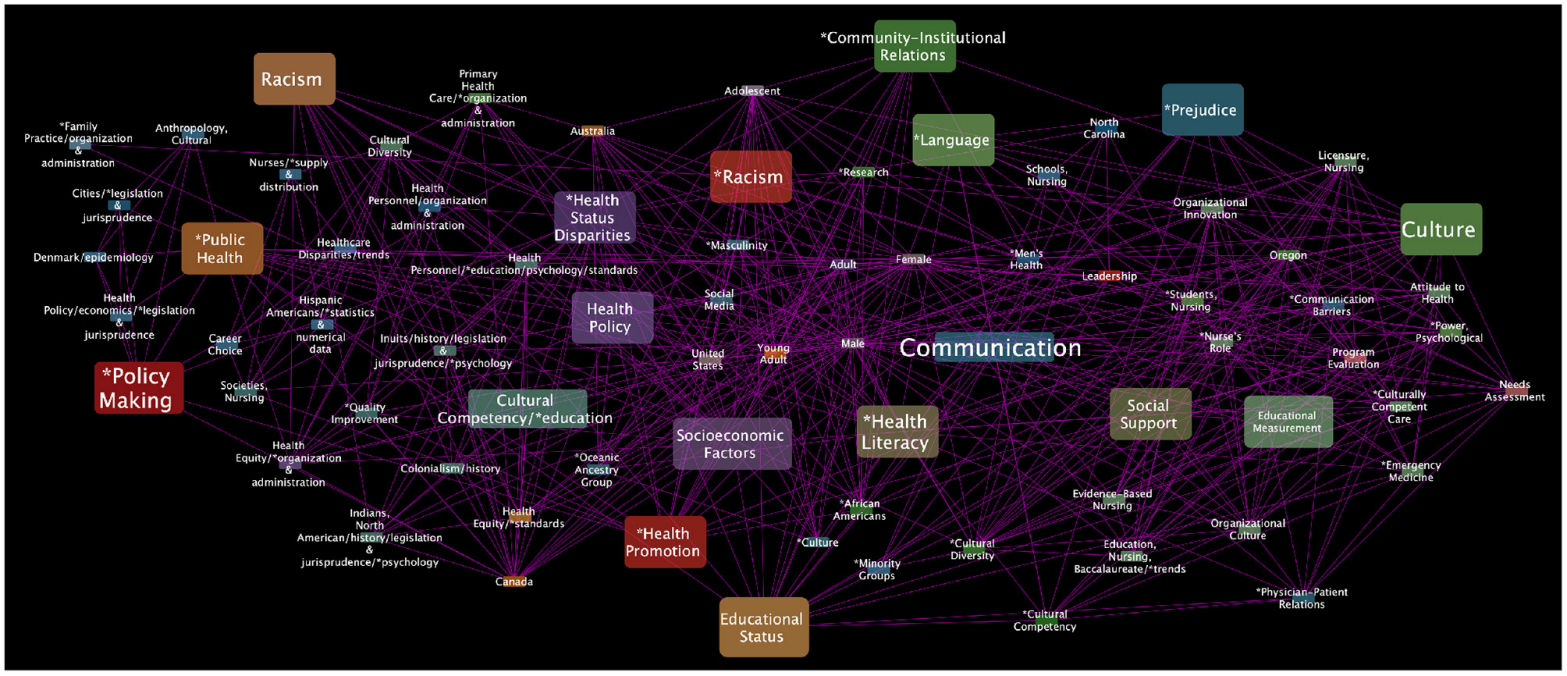
Health equity, Social determinants of health, and Culture MeSH terms subnetwork. This *HSDHC subnetwork* has 75 nodes and 519 edges.

**Figure 8:**
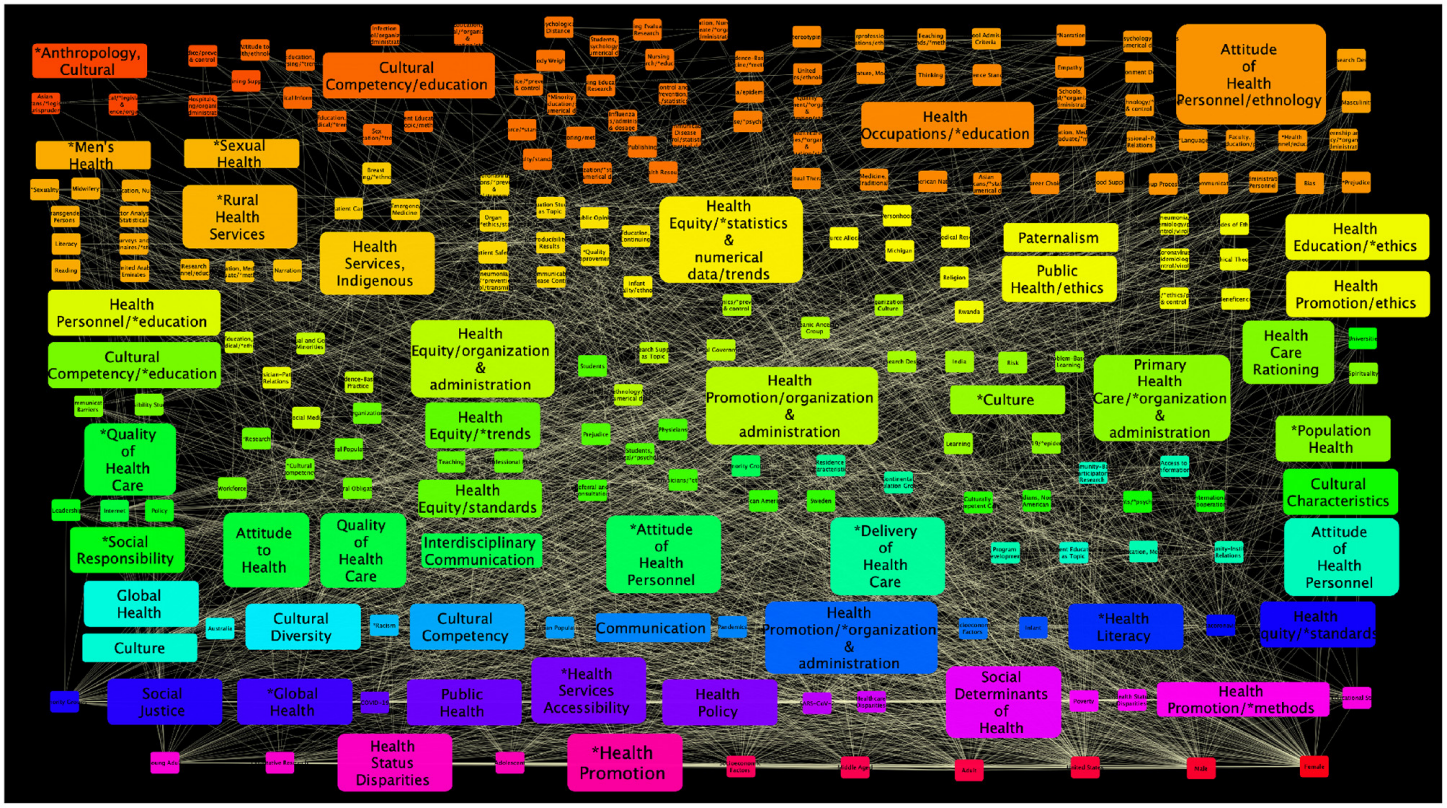
Health equity, Education, and Culture MeSH terms subnetwork. This *HEC subnetwork* has 232 nodes and 2,359 edges.

To continue extracting semantic context from the analyzed networks, three subnetworks were extracted from each of the main networks (Figures 3–5) to construct a theoretical discussion and visualize outstanding patterns based on the following MeSH terms and their first neighbors (see Figures 6–8):

- *Health equity, Vulnerable Populations* and *Culture* subnetwork (HVC subnetwork).
- *Health equity, Social determinants of health* and *Culture* subnetwork (HSDHC subnetwork).
- *Health equity, Education* and *Culture* subnetwork (HEC subnetwork).

These subnetworks and the underlying concepts will be further discussed in the context of the thematic analysis. This will considered in the corresponding heading in the Section 4 (Section 4.2).

## 4. Discussion

### 4.1. Semantic Relations

*Semantic networks* have been used to represent conceptual or referential relationships between concepts to generate knowledge *via* representation (63–65). In the present study, we have built several semantic networks over an ontology, that serves as a referential framework and is given by the Medical Subheading (MeSH) controlled and hierarchically organized vocabulary (23, 45, 46). These semantic networks were aimed to analyze the structural relationships behind concepts relevant to our understanding of how health equity and inequity phenomena arise in the different contexts of culture, education and other SDH. We believe that this approach is useful to analyze large literature corpora (such as the ones comprising the current health literature) and characterize the conceptual relationships of what is being discussed in a systematic and unbiased manner. Aside from setting the foundations for deeper explorations and critique, this approach allows us to discern biases and limitations, even fields of opportunity in the scholarly discussion of such relevant issues.

This is, in our view, a timely discussion. In recent times, the fact that health equity theory and practice are indeed subject to implicit and structural biases, has been highlighted repeatedly (66–69). This has been further noticed in the context of the ongoing COVID-19 pandemic (61, 62, 70–73). In what follows, we will briefly discuss some issues we find revealing and interesting regarding the current health professional literature trends on the relationships between concepts like *Health equity, Vulnerable populations, Social determinants of health, Culture, Trust, Health literacy* and *Education* as observed from analyzing the six semantic networks derived from the systematic literature search instances introduced in Section 2.6, and described in Section 3 (Sections 3.3.1 to 3.3.6), and in the related subnetworks as presented in Section 3.4.

We will first discuss, what we have learned by analyzing the semantic network obtained from the literature corpus of the joint search of *Health equity* [MeSH] and *Vulnerable populations* [MeSH] (Section 3.3.1). Even before actual analysis of the concepts and relationships associated. We were able to notice some particularities of the scholarly discussion on these matters.

First of all, the PubMed database grows in hundreds of thousands to millions of articles every year, in all fields of life sciences and biomedical topics (74). With this in mind, it seems astonishing that only 108 articles were found, with no more than 22 articles written on any given year regarding *Health equity* and *Vulnerable populations*. The second issue is that (as is often the case in the health research literature), the articles forming this corpus were written in a handful of countries, mostly corresponding to developed nations or emerging economies. These include the United States of America, Canada, Australia, United Kingdom, Brazil, Switzerland, Colombia, Lebanon, Germany, Sweden, France, Norway, Thailand, India, Netherlands, Spain, Denmark, Philippines, Argentina, Chile, China, Cuba, Ethiopia, Ireland, Italy, Jamaica, Kenya, Malawi, Mexico, Nepal, and Peru. The main contributors (as it can be seen in Figure 3B) are indeed the United States of America, Canada, and the United Kingdom whose socioeconomic conditions and the specificities of their health systems may drive important biases in the conclusions of their research making difficult to generalize some of the knowledge generated and presented in said articles, an issue that has been already documented in the context of the health professional literature (75–77), but also has been noticed in the context of health equity differences (5, 78–80).

Moving onto the results of the semantic network itself, one can notice that the distribution of *degree centralities* reveals interesting clues. Centrality degree—i.e., the number of relationships a given node-concept has in a semantic network—, has been recognized as a key indicator of the relevance of the concept to the overall conceptual picture of an issue as represented by the semantic network (81, 82). In this context, we have observed that some central concepts related to the situation of vulnerable populations with regards to health equity, such as *poverty, healthcare disparities*, and *age* are being recognized as such in the published health professional literature as indicated by their high degrees and priority rankings in the network. These terms are all in the Top 20 more *central* concepts, discussed in a larger number of publications in relation to different aspects of the health equity/vulnerable population studies. However, as previously commented in the Section 3, other terms that are intuitively relevant to this discussion are being somehow disregarded. For instance, the fact that *Social justice* and *Racism* are ranked in the 37th and 38th place for being connected with just 47 other concepts (out of 550 possible), a fact that may reveal important gaps in the literature, as has been known for some time (83–86).

Even more intriguing is the fact that *Sexual and gender minorities, Transients and migrants* and *Prisoners* admittedly some of the most vulnerable groups in relation to social equity are in the *periphery* of the semantic network, ranked in the 313, 337, and 474 out of 551 concepts, hence stressing even more their vulnerable role, not only in connection with healthcare, but even with respect to the health research literature (87–90). Perhaps, the most striking finding of our semantic network analysis of the relationship between *Health equity* and *Vulnerable populations* is the fact that *African Americans, American Indians or Alaska natives* and *Persons with mental disabilities* are not even explicitly mentioned as relevant concepts (i.e., these issues may be touched-upon in some of these articles, but no MeSH identifier has been recorded for these issues in any of the 108 articles analyzed to build this network.

In connection with the role that *Culture* and *Education* may play in the context of *Health equity* and *Vulnerable populations*, these are still rather peripheric concepts on this network. Hence, in the corpus of published health literature on health equity and vulnerable populations, topics such as *Attitude to health, Patient acceptance of healthcare, Culture* and *Cultural diversity* are not connected to the main concepts in this semantic network. This points out to the need, to bring the discussion on these important concepts into the mainstream health professional literature on health equity, integrating them appropriately.

Let us now analyze what we found in the semantic network built upon the search on *Health equity* and *Social determinants of health* encompassing 921 concepts as discussed in 254 published works. Following a similar pattern in the distribution of countries contributing to the scholarly discussion on these issues as it can be seen in Figure 4B, most of the literature comes from countries such as the United States of America, Canada, Australia and the United Kingdom, with some contributions by authors in Switzerland, Denmark, Egypt, Saudi Arabia, Sweden, Brazil, Thailand, Chile, Colombia, France, Germany, Mexico, Spain, Belgium, India, Kenya, Netherlands, Norway, Argentina, Chad, China, Ecuador, Ethiopia, Finland, Iran, Ireland, Israel, Italy, Malta, Peru, Poland, Portugal, and Swaziland. So we can notice the addition of countries with a broader scope of socioeconomic and cultural conditions. The number of yearly publications is a bit higher than in the previous network, but still rather small, with a maximum of 67 articles per year.

We observed that *some* terms associated with SDH, such as *Socioeconomic factors* and *Health status disparities* occupy relevant places in the semantic network (ranked 9 and 11 out of 921, respectively). However, other SDH are less connected in this network; *Social conditions* is ranked in the 63rd place, furthermore *Social support* which is a key structural determinant of health ranks in the 98th place with only 34 connections out of 920. In spite of its relevance as a relief factor to modulate SDH, *Social support* has been documented to be underrepresented in the specialized literature (91, 92). It has been discussed that publication biases regarding SDH may indeed obey cultural reasons, an issue that is central to the discussion of the role of culture and education in health equity (93–96).

The relationship between *Culture* itself and *Health equity* has been studied here, as it is presented in Section 3.3.3. Important concepts such as *Cultural diversity* and *Cultural competency* are well-represented concepts in this semantic network. These two concepts are indeed closely connected: embracing *Cultural diversity* helps healthcare providers to offer their services, recognizing the unique social, cultural and even linguistic features of their patients in the context of their populations (97, 98), this in turn leads to *Cultural competency* of the health systems, that is, the ability of such systems to provide care consistent with the values beliefs and behaviors of the patients (5, 99). However, other concepts such as *Empathy, Self-concept* and *Attitude to health/ethnology* are scarcely connected to the main discourse as reflected by their degree rankings (places 443, 530 and 564 out of 691 respectively). Calls to attention that, apparently, healthcare systems features are in a more common and centralized discussed in the current literature on health equity and culture than *individual* or *personal* concepts.

Such *personal* characteristics are indeed a central part in the establishment of *Trust* in the healthcare setting (100–104). The conceptual relationships around *Health equity* and *Trust* were also studied in detail here. In Section 3.3.4, we have presented some results of the analysis of this semantic network. As we already mentioned, such individual, even personal, *Human* features are scarcely discussed in the literature on *Healt equity*. We can notice, for instance, that the *Health equity - Trust* network is based on a smaller literature corpus of just 14 articles leading to a reduced network of only 140 concepts. Terms such as *Communication* and *Social support* are relatively well-connected in this network (ranks 8 and 12 out of 140, respectively), but related issues such as *Health communication* and *Communication barriers* are still peripheral concepts connected to just 9 and 7 out of 139 terms, ranking 125th and 134th. Since good communication is key to build proper trust relationships between patients and healthcare providers (105, 106), improving the discussion on these issues seems desirable.

Building up trust in healthcare systems needs improving communication channels (105, 107). To do this, often is needed to improve *Health literacy* (108, 109). The web of concepts related to the role played by *Health literacy* in *Health equity* was also explored, main results were outlined in Section 3.3.5. The network described therein is also a somewhat small network comprising 166 concepts connected by 1,670 semantic relationships taken from 27 articles. Aside from the search terms, few concepts pertinent to an actual discussion of the role that culture, education and trust play in the construction of health equity are at the core of this network. For instance, the MeSH term *Health knowledge, attitudes, practice* that in our view would be quite relevant is indeed placed 112 out of 166 concepts in terms of conceptual connectivity. Furthermore, most of the discussion along these lines refer to the knowledge, attitudes and literacy *of healthcare workers* to attain health equity. While enormously important, healthcare workers and providers are just one side of the story. The discussion on health literacy, attitudes to knowledge and culture from the standpoint of the patients and their families has been largely disregarded (110–112).

These issues are indeed closely related to our findings in the context of the *Health equity - education* axis, as presented in Section 3.3.6. In contrast with the two previously discussed conceptual networks, this one is much larger (1,673 concepts) and *denser* (21,952 semantic relationships) with information coming from 420 published research works. *Health promotion* appears in the core of the network (ranked 10 out of 1,673 concepts), something we consider to be positive. Also relatively central to the discussion are concepts like *Educational status, Health literacy* and *Cultural competency* (ranked 21, 53, and 70, respectively). Though somewhat less connected, *Patient education as topic* is still within the top100 (rank 94 out of 1,673) more relevant terms. We believe that some improvement can be made in this regard, in particular since patient education has been described as instrumental to achieve health equity (113–115).

### 4.2. Thematic Analysis

To deepen on the discussion about focal issues, we have performed *thematic analysis* of the literature corpus using Atlas-ti over the associated domains in the semantic network. In this regard, we can deliberate upon the following matters:

Regarding vulnerable populations, we examined the specific connections of the *Culture* MeSH terms in a subnetwork of the semantic network in Figure 3. As you can see in the resulting (Figure 6), some main terms are directly or indirectly connected with *Culture* MeSH term, some of them are highlighted (larger node size) such as *Hispanic Americans, Emigrants and Immigrants, Ethnic groups, Minority groups* and *Continental populations groups*. However, there are other critical terms related to vulnerable population that are not connected within the Culture MeSH term subnetwork (composed of 490 MeSH terms), such as, *Homeless persons, Prisoners, Disabled persons, Refugees, Rural population, Intellectual disability, Disabled persons, Sexual and gender minorities, Terminally ill people, People suffering violence*. In light of the thematic analysis carried out with Atlas.ti, they seem to be important social and cultural determinants that can determine some inequities in health. For example, people experiencing homelessness or vulnerable housing are often marginalized and are known to face barriers to accessing appropriate healthcare services (9). Although changes have been recommended in the complex health systems, so that it should be more equitable, more sensitive and empathic, and more informed about the traumatic situations experienced by homeless people, barriers related to cultural aspects are barely mentioned in those key documents (116–118). Similar patterns can be seen in the studies of other conditions of vulnerability and adverse circumstances that apparently seem disconnected from their own cultural aspects or they seem irrelevant for health equity (119).

Regarding the Health equity, Education and Culture subnetwork (see Figure 8) the terms *Health promotion, Health services accessibility*, and *Social justice* are highly interconnected and related to other key terms such as *Cultural diversity, Cultural competence, Attitude to Health, Interdisciplinary communication, Health policy* and *Health literacy*. However, in this triad of terms, some no less important but not directly connected to culture have been excluded (the subnetwork is composed of 1440 MeSH terms), such as: *Health behavior, Cooperative behavior, Social support, Social stigma, Health knowledge, attitudes and practice, Social Class, Education Medical, Community participation, Life style, Consumer health information, Decision making, Self-management, Quality of life, Social change, Personal satisfaction, Social welfare, Motivation, Interpersonal relations, Professional competence, Social environment, Social conditions, Health services needs and demand, Treatment outcome, Social skills, Resilience, Social values, Social norms, Life expectancy*.

The thematic analysis also shows that are important social and cultural determinants that can influence some inequities in health. Health literacy has been a particularly prominent issue on the political, academic and scientific discourse on equity in health. The World Health Organization has established an urgent mandate for public policy action on health literacy as a key pillar for achieving health equity worldwide (120). In the reviewed documents, health literacy is presented as a universal challenge associated with wellbeing, access to healthcaere and improved health outcomes (121–123). However, this concept is interrelated with others that apparently are not related to culture and health equity. For example, the health literacy community movement driving for social change toward empowerment and health equity is related to some concepts like *Public health, Social change*, or *Social support*. But, the literature mentions that social movements are developed to impact health by generating changes related to cultural and social norms (124). Also, other documents mention that health literacy is necessary to make appropriate decisions regarding health. And again points out that some cultural factors contribute to reducing health inequities in this regard. The scholarly discussion on these issues is hence still far from being conclusive.

Other prominent social determinants of health derived from the thematic analysis of literature were connected to social and cultural responses for health equity. Not only the health care sector, but also, the education sector, administrators, financial systems, reimbursement mechanisms, industry, community centers, civil society groups, social networks, political organizations, even artists or cultural workers among others play a critical role in creating conditions for intersectoral collaboration and distributing resources that promote health equity (125–134). Theses roles appear, indeed, disconnected from the main discourse on the matters.

Finally, we explored the subnetwork of Health equity, Social Determinants of health and Culture subnetwork in the light of the thematic analysis. Some relevant terms emerged such as: *Socioeconomic factors, Social support, Cultural competency, Health promotion, Educational status, Health literacy, Comunication, Health policy, Language, Racism* and *Prejudice* (see Figure 7). However, some MeSH terms were less common in the studies reviewed and are not directly connected to the culture term (around 846 MeSH terms in the subnetwork). For example, *Poverty, Housing, Residence characteristics, Income, Environment, Food security, Race factors, Adverse childhood experiences, Social discrimination, Public assistance, Employment, Healthcare financing, Language, Health services, Social capital, Social welfare, Healthy lifestyle, Social networking, Social segregation, Urbanization, Social Isolation*, among others. Many of these overlap in the same individuals or communities, exacerbating their vulnerability and the health inequities (135). The characterization of SDH is critical to implementing actions that are more inclusive of and more sensitive to the different needs of the population as the WHO has instructed in many regions of the world, especially for disadvantaged sectors of society (14). However, we again identify some research gaps in terms of social determinants and cultures that can be explored in the future to help understand their interaction with health equity. We have presented and discussed only a few instances of the many connections and biases that can be found in the healthcare literature about health equity and culture. By resorting to the generated searches and resources available here and in other studies, the interested researchers may indeed discover many more instances and relationships relevant to these important yet somehow understudied issues.

### 4.3. Study Assumptions, Scope, and Limitations

As previously mentioned, the current study is founded on several basic assumptions that will shape the scope and present some limitations. The main analysis is somehow constrained by the use of the MeSH term classifiers. While this is an excellent method for identifying major research topics, emerging or potentially interesting topics may not be easy to spot, still presents an incomplete picture (33).

Although we carried out a complementary search in other multidisciplinary databases (LILACS and DOAJ) and both offer some kind of controlled vocabulary, the results differ significantly, though the main conclusions still hold. This happens because in some archiving schemes data curation is often limited to a simple thesaurus of keywords or concepts; which can also affect the correct interpretation of the search, and the reproducibility or comparability of the results.

Another issue that may be considered a limitation is that we used open access databases, which can also contribute to not achieving a complete search. However, the use of restricted access databases (some of which are behind expensive “paywalls”) would bring another set of limitations, mostly regarding accession bias. Furthermore, restricted access to health information contributes to deepening the gaps and increasing health inequities.

Also, we are aware that different search systems may give rise to different results even when the same query has been employed, as these systems have different indexing methods, data presentation, and curation methods (136). Retrospective coverage of the controlled vocabulary may be limited, for instance. Thus, it may be difficult to quantify the quality of such controlled vocabulary as their features are diverse.

Additionally, as already mentioned, the choice of MeSH classifiers as the basis for the semantic network analysis introduced a number of assumptions (see Section 2.6).

Other methodological constraints arise from the use of automated or semi-automated analysis tools. The use of software such as Cytoscape or Atlas.ti to conduct data analysis also has a limitation related to the decontextualization of the findings, which can result in data interpretation weaknesses. However, the statements of researchers from different fields of knowledge concerning the advantages and disadvantages of the software used have diminished over the time with the evidence of its usefulness for analysis (137).

## 5. Final Remarks

This work aimed to characterize, at a large scale, how social and cultural determinants may interact with health inequity and the interrelationships among them in different populations and diverse contexts. To this end, we have introduced semantic networks as a theoretical framework and methodological tool to carry out this analysis in a comprehensive, minimally-biased manner. We have built semantic models based on an ontology representation given by the Medical Subheading (MeSH) identifiers as developed by the National Library of Medicine of the United States of America and implemented our network construction based on a set of preselected searches in the PubMed database. Since MeSH terms were developed to be general purpose identifiers and being PubMed the most comprehensive database of academic publications in medicine and related topics; we believe that using these resources, though not ideal, is the least-biased and more comprehensive automated approximation to analyze the scholarly literature on these issues.

Our semantic network approach confirms the central role of some concepts in the academic discussion on health equity and culture, in the context of vulnerable populations, taking into account their SDH and how trust may arise in the different circumstances of health literacy and education. However, we have also found some biases and under-representation of several relevant concepts, likely influenced by the fact that the academic literature is both relatively scarce and produced in a few countries. Most of these countries are actually developed or emerging economies characterized by firmly established trends in their health systems. By pointing out such biases and sub-represented concepts in the discussion, it is possible to identify areas of opportunity for further academic development. Our view as presented here is of course, of a rather general and broad scope. However, the curated literature corpora, the semantic networks built and their statistical and topological structure analysis provided as Supplementary Materials may constitute a useful resource to navigate the full body of literature on these issues. Further insight was derived by considering additional data sources and by performing thematic analysis of discourse. However, perhaps the main *conclusion* is that there is still a long way to go toward a full scholarly representation of health equity and its relation to culture, with their many facets and complexities.

## Data Availability Statement

The original contributions presented in the study are included in the article/Supplementary Material, further inquiries can be directed to the corresponding author/s.

## Author Contributions

EH-L and MM-G: conceptualization, methodology, software, supervision, and validation. EH-L, MM-G, and JMVC: formal analysis and writing—original draft preparation, review, and editing. All authors read and approved the final manuscript.

## Conflict of Interest

The authors declare that the research was conducted in the absence of any commercial or financial relationships that could be construed as a potential conflict of interest.

## Publisher's Note

All claims expressed in this article are solely those of the authors and do not necessarily represent those of their affiliated organizations, or those of the publisher, the editors and the reviewers. Any product that may be evaluated in this article, or claim that may be made by its manufacturer, is not guaranteed or endorsed by the publisher.
